# Influenza Vaccine Effectiveness Against Influenza A(H1N1)pdm09‐Associated Hospitalizations With SARI in Beijing, China, in the 2024–2025 Season

**DOI:** 10.1111/irv.70248

**Published:** 2026-03-12

**Authors:** Chunna Ma, Ying Sun, Jiaxin Ma, Yingying Wang, Xiaodi Hu, Ying Shen, Li Zhang, Jiaojiao Zhang, Lu Zhang, Wei Duan, Jia Li, Quanyi Wang, Daitao Zhang, Peng Yang

**Affiliations:** ^1^ Beijing Research Center for Respiratory Infectious Diseases Beijing China; ^2^ Beijing Key Laboratory of Surveillance, Early Warning and Pathogen Research on Emerging Infectious Diseases Beijing Center for Disease Prevention and Control Beijing China; ^3^ Institute for Infectious Disease and Endemic Disease Control Beijing Center for Disease Prevention and Control Beijing China; ^4^ School of Public Health Capital Medical University Beijing China

**Keywords:** China, hospitalization, influenza, test‐negative design, vaccine effectiveness

## Abstract

**Background:**

In the 2024–2025 season, influenza activity returned to pre‐COVID‐19 pandemic levels, with A(H1N1)pdm09 as the predominant subtype. Reassessment vaccine effectiveness (VE) in hospitalized patients is critical to inform potential adjustments to vaccination policy.

**Methods:**

Using a test‐negative design, we evaluated the VE of the seasonal influenza vaccine during the 2024–2025 season against A(H1N1)pdm09‐associated hospitalizations with severe acute respiratory infections (SARI) in Beijing, China, from December 9, 2024 to February 9, 2025. VE was estimated by comparing the odds of influenza vaccination between case‐patients (those testing positive for A(H1N1)pdm09) and controls (influenza test‐negative patients), applying inverse‐propensity‐to‐be‐vaccinated weights.

**Results:**

The analysis included 1608 hospitalized SARI patients (17.4% tested positive for A(H1N1)pdm09; 13.8% were vaccinated overall). The adjusted VE against A(H1N1)pdm09‐associated hospitalizations was 18.8% (95% CI: 0.9% to 33.6%) overall. Specifically, the VE was 45.4% (95% CI: 10.0% to 67.1%) in children aged 0.5–17 years, 43.8% (95% CI: −20.8% to 76.1%) in adults aged 18–59 years, and 8.6% (95% CI: −15.8% to 27.9%) in older adults ≥ 60 years. Among individuals with chronic conditions, the VE was 18.5% (95% CI: −2.3% to 35.2%) and was higher among those with chronic respiratory conditions (40.6%, 95% CI: −1.7% to 65.7%).

**Conclusions:**

During the 2024–2025 season in Beijing, the overall VE against A(H1N1)pdm09‐associated SARI hospitalizations was suboptimal. However, moderate VE was observed among children and younger adults, while limited effectiveness was seen in the elderly. Optimizing influenza vaccination strategies, including the introduction of high‐dose or adjuvanted vaccines to enhance immune response in older adults, is crucial to alleviate influenza‐associated hospitalizations.

## 1 Introduction

In the aftermath of the COVID‐19 pandemic, influenza has returned to its prepandemic epidemic patterns. The 2024–2025 influenza season in the Northern Hemisphere is marked by an initial dominance of influenza A, followed by either a codominance of A and B or a predominance of B [1, 2]. In China, and specifically in Beijing, the 2024–2025 influenza season (commenced in early November 2024 and peaked in early January 2025), with a dominance of A(H1N1)pdm09 viruses [3]. These viruses were all grouped within clade 6B.1A.5a.2a [4], a clade that was also widespread in Europe (by Week 6 of 2025) and in North America (by Week 52 of 2024) [5, 6]. Shifts in viral clades are critical determinants of influenza vaccine effectiveness (VE), as discrepancies between vaccine strains and circulating strains can significantly diminish VE. Consequently, the circulation of this specific A(H1N1)pdm09 clade during the 2024–2025 season necessitates an urgent and targeted assessment of VE to confirm the protective efficacy of current vaccines against this predominant strain.

Seasonal influenza imposes a significant socioeconomic burden, manifesting in 3 to 5 million severe cases and 290,000 to 650,000 respiratory‐related deaths annually [7]. Populations at elevated risk for severe disease, complications, and mortality include older adults (aged 65 years and above), pregnant women, young children, and individuals with chronic comorbidities or immunosuppression [7, 8, 9]. Mitigating these severe outcomes—particularly hospitalizations due to severe acute respiratory infection (SARI)—is heavily dependent on influenza vaccination, which is recognized as an effective strategy for preventing influenza and its serious complications [10]. Nevertheless, the success of vaccination programs is intrinsically linked to VE, which directly influences the degree to which vaccines can reduce severe illness and hospitalizations within at‐risk populations.

VE is not a constant metric and is subject to variation based on vaccination coverage. As coverage fluctuates across different demographic groups and temporal periods, the degree of indirect protection, or herd immunity, also varies, potentially leading to significant alterations in VE estimates in observational studies. Since 2007, Beijing's influenza vaccination policy has offered free vaccines to adults aged 60 years and older, as well as to students aged 6 to 17 years, thereby enhancing coverage within these target populations [11]. In the 2022–2023 season, vaccination coverage was reported to be 25.89% among individuals aged 6 to 18 years, 12.08% among those aged 60 years and older, and 8.75% overall [12]. The city's overall vaccination rate, measured as 6.5% among outpatient ILI cases during the 2021–2022 season [13], was higher than China's national average of 3.16% in 2020–2021 [14] but lower than the rates reported in European countries (20%, 2020–2021) and the United States (45.9%, 2020–2021) [15, 16].

The most recent evaluation of influenza VE against hospitalization in Beijing, China, was conducted 9 years prior [17]. Since that time, the circulating A(H1N1)pdm09 clades have undergone evolution, and the immunity profiles of the population may have shifted—both of which are factors that can significantly influence VE. Previous research has highlighted the influenza‐associated hospitalization rate in Beijing hospitalization rate (38–39 per 100,000) and the 16.6% proportion of SARI cases attributed to influenza [17], emphasizing the pressing need to update VE data. Accurate and current VE estimates are crucial for informing policy modifications, such as optimizing vaccination prioritization and enhancing coverage, as well as refining vaccination strategies to mitigate influenza‐related hospitalizations.

To address this critical gap, the primary research question of this study was to estimate the VE of influenza vaccines against influenza A(H1N1)pdm‐associated SARI hospitalizations in Beijing, China, in the 2024–2025 season. Additionally, we sought to examine VE in patients with chronic comorbidities (cardiovascular, cerebrovascular, and respiratory diseases), as these high‐risk groups exhibit unique vaccination response profiles.

## 2 Methods

### 2.1 Study Design, Participants, and Laboratory Test

A test‐negative control design was employed to assess the influenza VE against influenza‐associated hospitalized SARI cases at the sentinel hospitals within the Multi‐Pathogen Surveillance System in Beijing, China. Hospitalized SARI was defined as inpatients presenting the following clinical symptoms within 48 h of admission: acute onset, fever (≥ 38.0°C), cough, and a disease duration of ≤ 10 days. The Multi‐Pathogen Surveillance System, established in 2024 to address the urgent need for real‐time monitoring and early warning [18], comprises 19 sentinel hospitals and 16 network laboratories across Beijing. Within this framework, trained clinicians collected data and 10 respiratory tract specimens weekly from hospitalized SARI patients in sentinel hospital wards, including the respiratory ward, pediatric internal medicine ward, critical care medicine ward, infection department ward, emergency department intensive care unit (ICU), and the department of critical care medicine at each sentinel hospital. The specimens were transported to designated network laboratories in viral transport medium at 4°C for nucleic acid detection of the influenza virus, SARS‐CoV‐2, and other common respiratory pathogens using reverse transcription polymerase chain reaction (RT‐PCR).

The term “peak week” denotes the calendar week within the 2024–2025 influenza season that exhibited the highest weekly influenza positivity rate, as determined by virological surveillance data. The influenza epidemic period is subsequently defined as the series of consecutive weeks during which the weekly influenza positivity rate reached or exceeded 40% of this peak value [19]. According to this definition, the epidemic period identified in our study spans from Week 50 of 2024 (December 9th to 15th) to Week 6 of 2025 (February 3rd to 9th).

**FIGURE 1:**
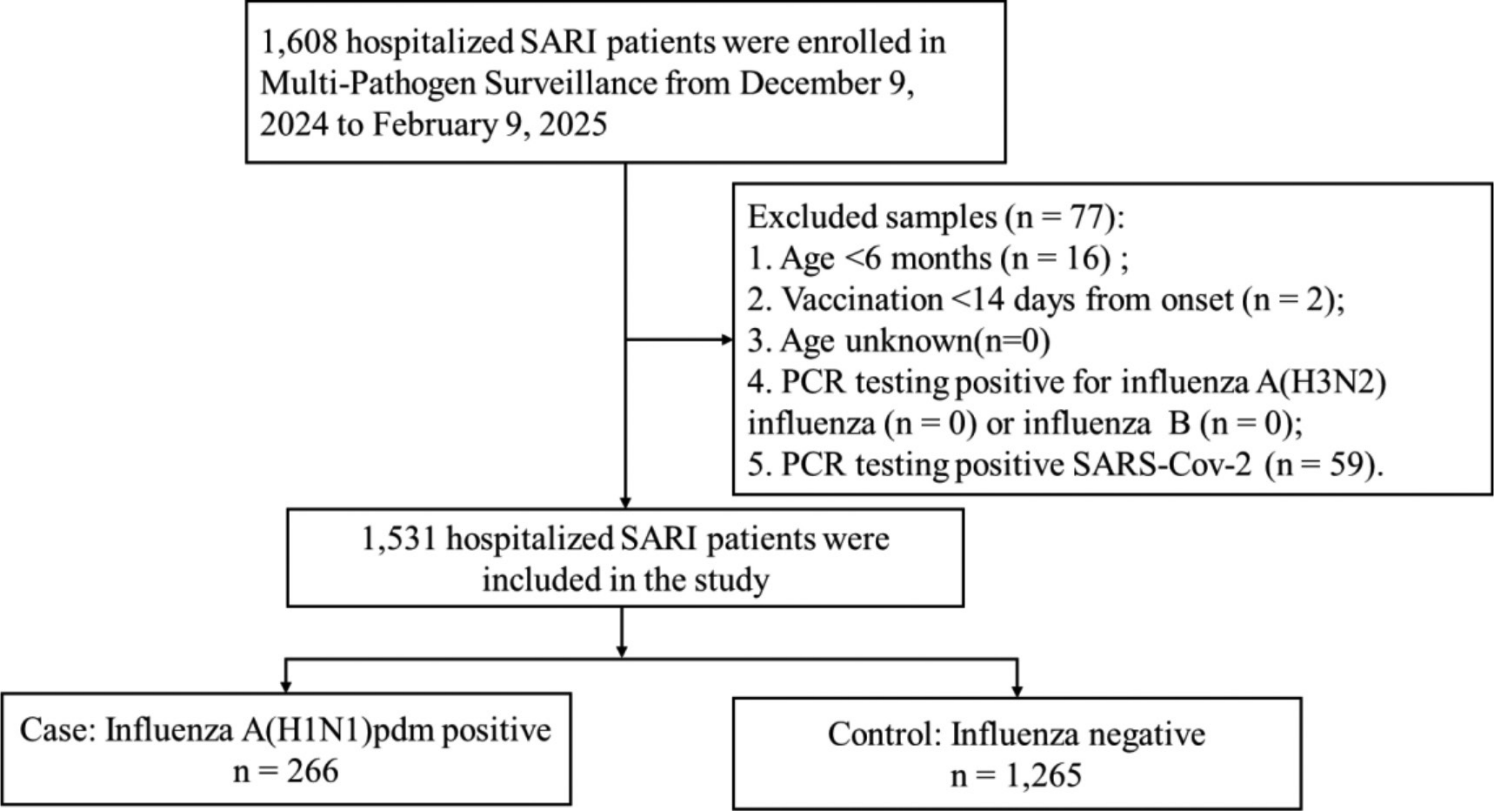
Flowchart of subject enrollment in the test‐negative design study for estimating influenza vaccine effectiveness among hospitalized patients with severe acute respiratory infection (SARI) in Beijing, China, in the 2024–2025 season.

Currently, the influenza vaccines authorized for use in China include trivalent inactivated influenza vaccines (IIV3), quadrivalent inactivated influenza vaccines (IIV4), and trivalent live‐attenuated influenza vaccines (LAIV3), which are exclusively applicable to individuals aged 3 to 17. Adjuvanted influenza vaccines and high‐dose influenza vaccines are not yet available [20]. Given that the influenza vaccines approved in China are only indicated for individuals aged 6 months and older, children under 6 months were excluded from the study. Furthermore, considering that a minimum of 14 days is required to achieve a protective antibody level following influenza vaccination, individuals vaccinated within 14 days prior to the onset of symptoms were also excluded. During the 2024–2025 influenza season, the A(H1N1)pdm09 was the predominant strain, with only sporadic detections of A(H3N2) and B influenza viruses. Consequently, the analysis focused exclusively on the VE against A(H1N1)pdm09, and participants testing positive for A(H3N2) and B lineage viruses were excluded. Additionally, individuals with SARS‐CoV‐2 infection were excluded to mitigate potential confounding effects related to the association between influenza and COVID‐19 vaccine administration, which could lead to a downward bias in influenza VE estimates [21, 22]. The specific inclusion and exclusion criteria employed are presented in Figure 1.

### 2.2 Data Collection

Epidemiological data from sampled patients were systematically collected by healthcare professionals at sentinel hospitals using a standardized electronic questionnaire. This questionnaire included demographic details (age and sex), underlying medical conditions and clinical information (visiting date and swab collection time). Medical conditions included asthma, chronic tracheitis or bronchitis, chronic obstructive pulmonary disease (COPD), hypertension, coronary heart disease, diabetes, hyperlipidemia, HIV/AIDS, tumors, and cardiovascular hematologic, cerebrovascular, renal, hepatic, neurological, and immune system conditions. Vaccination status was obtained from the Beijing Management System of Information on Immunization Program. By scanning vaccines' unique identifiers, this system enables full‐process traceability and automated management throughout vaccine warehousing, outbound logistics, and inoculation, while automatically capturing key details (e.g., batch and expiration date). At inoculation, it records real‐time, accurate data including date, injection site, and vaccinee.

### 2.3 Statistical Analysis

Data entry was performed using EpiData software (version 3.1; The EpiData Association, Odense, Denmark), and statistical analyses were conducted using R software (version 4.4.1). Demographic and clinical characteristics were summarized by vaccination status and case–control status using frequencies and percentages. All statistical tests were two‐sided, with *p* < 0.05 considered significant. VE was calculated as [1‐adjusted odds ratio (OR)]*100%. Multivariable logistic regression models were employed to estimate ORs comparing vaccination status between cases and controls. These models were adjusted for confounders, including study site, sex, age, calendar time, chronic conditions, and the interval between symptom onset and sampling. In subgroup analyses, variables with *p* < 0.1 in univariable analyses were further included in multivariable models. Age and calendar time were modeled using natural cubic spline variables. Inverse‐propensity‐to‐be‐vaccinated weights (IPVWs) were estimated and were subsequently applied in logistic regression models to address additional imbalances between vaccinated and unvaccinated group [23, 24]. The selection of variables for the IPVW adjustment was informed by both empirical differences in distribution between the groups and epidemiological evidence underscoring their significance as potential determinants of vaccination willingness.

VE against influenza A(H1N1)pdm09‐associated hospitalization was assessed by age group (0.5–17 years, 18–59 years, and ≥ 60 years), presence of chronic conditions (any and none), respiratory conditions (any and none), and cardiovascular and cerebrovascular conditions (any and none). VE was also evaluated based on vaccination combinations (unvaccinated, vaccinated in 2023–2024 season only, vaccinated in 2024–2025 season only, and vaccinated in both season) and the time interval between vaccination and onset (unvaccinated, 14–89 days, and ≥ 90 days).

## 3 Result

### 3.1 Influenza Virus Activity

Between December 9, 2024, and February 9, 2025, a total of 1608 hospitalized patients with SARI were enrolled at sentinel hospitals participating in the Multi‐Pathogen Surveillance program. Their specimens were subsequently tested in collaborating laboratories. Of these patients, 77 were excluded for not meeting the inclusion criteria (details provided in Figure 1). Among the remaining 1531 hospitalized SARI patients, 266 (17.4%) tested positive for influenza A(H1N1)pdm09 and were assigned to the case group, while 1265 (82.6%) influenza‐negative patients were assigned to the control group (Figure 1).

**FIGURE 2:**
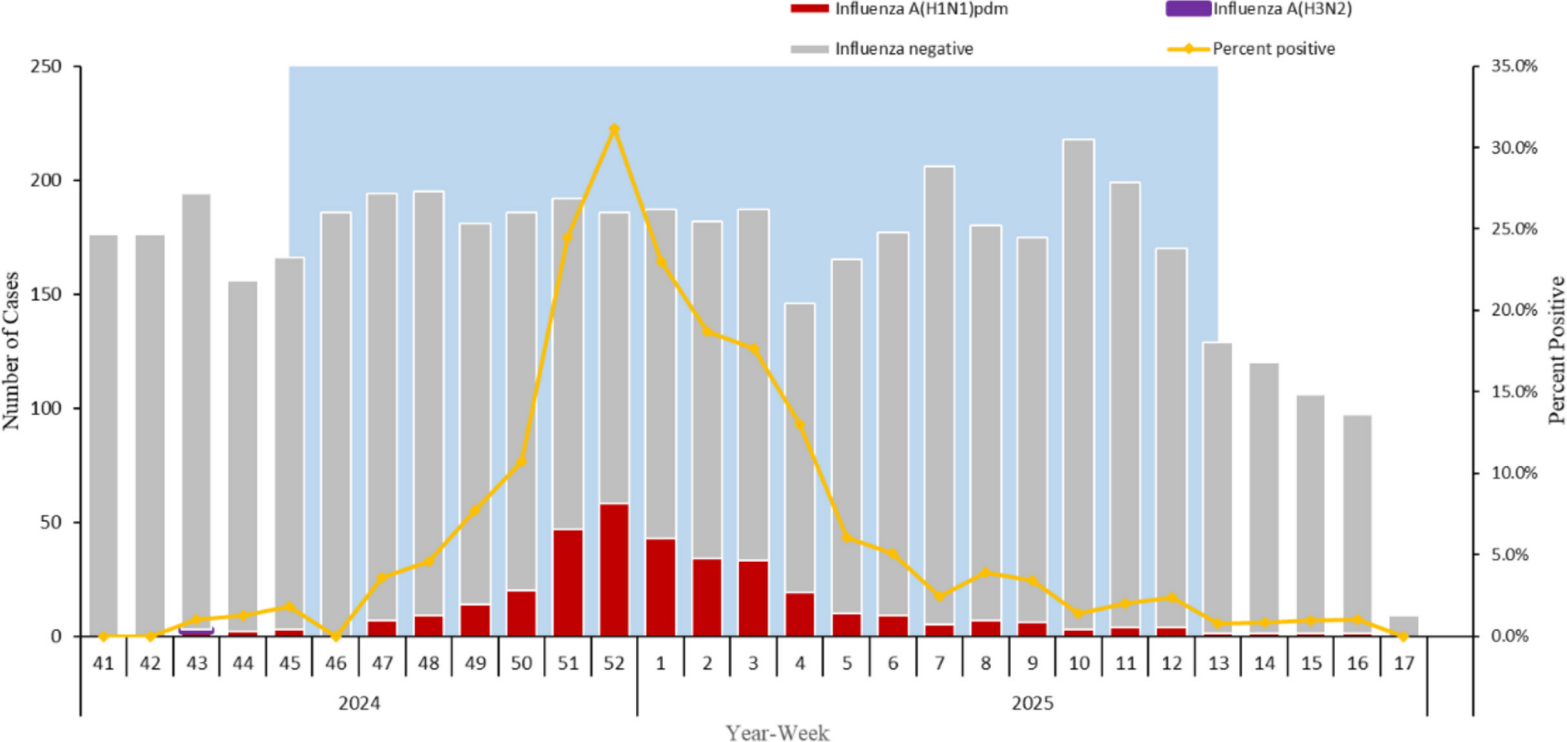
Timeline of recruitment of hospitalized patients with severe acute respiratory infection (SARI) testing positive or negative for influenza virus by type/subtype in Beijing, China, in the 2024–2025 season. Only one H3N2‐positive specimen was detected in Week 43, 2024; no Influenza B‐positive specimen was detected.

Prior to Week 42 of 2024, no hospitalized SARI patients tested positive for influenza. The positive rate for the influenza virus began increasing from Week 47, 2024, peaking in Week 52 at 31.2% (Figure 2).

### 3.2 Participant Characteristics

**FIGURE 3:**
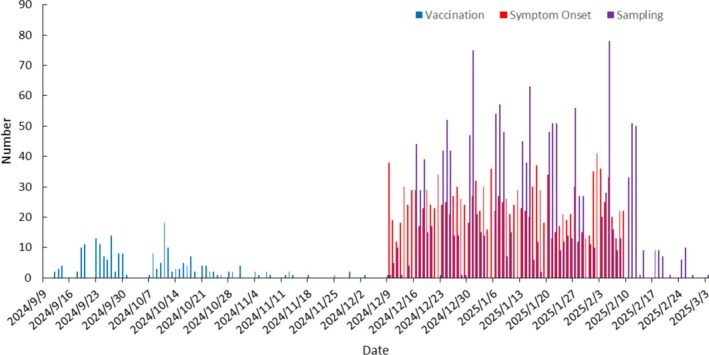
Timeline of vaccination, symptom onset, and sampling counts among hospitalized patients with severe acute respiratory infection (SARI) in Beijing, China, in the 2024–2025 season.

The vaccination dates for the study participants spanned from September 12 to December 23, 2024. Regarding the vaccination‐to‐onset interval, the minimum interval was 19 days, the maximum was 147 days, and the mean ± standard deviation was 93.98 ± 25.43 days. The earliest recorded onset and sampling dates were both December 9, 2024. For the onset‐to‐sampling interval, the minimum interval was 0 days, the maximum was 28 days, and the median (interquartile range) was 5 days (3–8 days) (Figure 3).

The positive rate for influenza A(H1N1)pdm09 was significantly higher in male than in female (19.3% vs. 15.1%; *p* = 0.033). The median ages of the influenza case group and the control group were 70.0 years (interquartile range [IQR]: 24.0) and 61.0 years (IQR: 63.0), respectively. By age group, the highest positive rate was observed in older adults aged 60 years and above (21.6%), followed by younger adults aged 18–59 years (16.7%), and then children aged 0.5–17 years (8.9%) (*p* < 0.001). The positive rate was significantly higher among individuals with chronic conditions compared to those without (19.5% vs. 11.9%; *p* < 0.001). Specifically, the positive rate was higher among patients with cardiovascular and cerebrovascular conditions compared to those without these conditions (22.8% vs. 14.3%; *p* < 0.001), but no significant difference was observed in the positive rate among patients with or without chronic respiratory diseases (19.1% vs. 17.1%; *p* = 0.493). The positive rate was higher for patients with onset in December (23.1%) and January (17.1%). By swab collection timing, the positive rate was highest (20.9%) when specimens were collected 0–3 days after onset, followed by ≥ 8 days (16.0%) and 4–7 days (15.4%) (*p* = 0.039) (Table 1).

**TABLE 1:** Characteristics of hospitalization patients with severe acute respiratory infection (SARI) by influenza vaccination and test status during the 2024–2025 season in Beijing, China.

Characteristics	Total, no. (col. %) *N* = 1531	Influenza vaccination status			Influenza A(H1N1)pdm test result		
		Vaccinated, no. (row %) *N* = 212	Unvaccinated, no. (row %) *N* = 1319	*p*	Positive, no. (row %) *N* = 266	Negative, no. (row %) *N* = 1265	*p*
**Group**							0.034
Unvaccinated	1319 (86.2)	0 (0.0)	1319 (100.0)		240 (18.2)	1079 (81.8)	
Vaccinated	212 (13.8)	212 (100.0)	0 (0.0)		26 (12.3)	186 (87.7)	
**Site**				< 0.001			0.620
Urban	544 (35.5)	51 (9.4)	493 (90.6)		91 (16.7)	453 (83.3)	
Rural	987 (64.5)	161 (16.3)	826 (83.7)		175 (17.7)	812 (82.3)	
**Sex**				0.479			0.033
Male	836 (54.6)	111 (13.3)	725 (86.7)		161 (19.3)	675 (80.7)	
Female	695 (45.4)	101 (14.5)	594 (85.5)		105 (15.1)	590 (84.9)	
**Age (years), M (IRQ)**	63.0 (60.0)	42.5 (67.0)	63.0 (47.0)	< 0.001	70.0 (24.0)	61.0 (63.0)	< 0.001
**Age group**				< 0.001			< 0.001
0.5–17 years	403 (26.3)	101 (25.1)	302 (74.9)		36 (8.9)	367 (91.1)	
18–59 years	276 (18.0)	7 (2.5)	269 (97.5)		46 (16.7)	230 (83.3)	
≥ 60 years	852 (55.6)	104 (12.2)	748 (87.8)		184 (21.6)	668 (78.4)	
**Any chronic condition**				0.038			< 0.001
None	436 (28.5)	73 (16.7)	363 (83.3)		52 (11.9)	384 (88.1)	
Any	1095 (71.5)	139 (12.7)	956 (87.3)		214 (19.5)	881 (80.5)	
**Respiratory condition**				0.494			0.493
No	1343 (87.7)	189 (14.1)	1154 (85.9)		230 (17.1)	1113 (82.9)	
Yes	188 (12.3)	23 (12.2)	165 (87.8)		36 (19.1)	152 (80.9)	
**Cardiovascular and cerebrovascular condition**				0.030			< 0.001
No	974 (63.6)	149 (15.3)	825 (84.7)		139 (14.3)	835 (85.7)	
Yes	557 (36.4)	63 (11.3)	494 (88.7)		127 (22.8)	430 (77.2)	
**Month of symptom onset**				0.492			< 0.001
December 2024	571 (37.3)	75 (13.1)	496 (86.9)		132 (23.1)	439 (76.9)	
January 2025	713 (46.6)	97 (13.6)	616 (86.4)		122 (17.1)	591 (82.9)	
February 2025	247 (16.1)	40 (16.2)	207 (83.8)		12 (4.9)	235 (95.1)	
**Interval between onset and sampling**				0.022			0.039
0–3 days	492 (32.1)	54 (11.0)	438 (89.0)		103 (20.9)	389 (79.1)	
4–7 days	559 (36.5)	94 (16.8)	465 (83.2)		86 (15.4)	473 (84.6)	
≥ 8 days	480 (31.4)	64 (13.3)	416 (86.7)		77 (16.0)	403 (84.0)	

The overall vaccination rate was 13.8% (212/1531). Among the 212 vaccine recipients, the predominant vaccines administered were trivalent inactivated influenza vaccines (IIV3) and quadrivalent inactivated influenza vaccines (IIV4), with only one case receiving trivalent live‐attenuated influenza vaccines (LAIV3). The vaccination rate was significantly higher in rural areas compared to urban areas (16.3% vs. 9.4%; *p* < 0.001). By age group, the highest vaccination rate was observed in children aged 0.5–17 years (25.1%), followed by older adults aged ≥ 60 years (12.2%), and younger adults aged 18–59 years (2.5%) (*p* < 0.001). Individuals with chronic conditions had a lower vaccination rate than those without (12.7% vs. 16.7%; *p* = 0.038). Specifically, patients with cardiovascular and cerebrovascular diseases had a lower vaccination rate than those without such conditions (11.3% vs. 15.3%; *p* = 0.030). No significant difference in vaccination rate was found between patients with and without chronic respiratory diseases (12.2% vs. 14.1%; *p* = 0.494), nor between males and females (13.3% vs. 14.5%; *p* = 0.479) (Table 1).

### 3.3 Influenza Vaccination Effectiveness

Among hospitalized patients with SARI, 18.2% of unvaccinated individuals tested positive for influenza, compared to 12.3% of those who were vaccinated (Table 1). The vaccination rates in the case group were lower than those in the control group (9.8% vs. 14.7%; *p* = 0.034). The unadjusted VE against influenza A(H1N1)pdm09‐associated hospitalization with SARI was 37.2% (95% CI: 4.7% to 60.1%) overall. After adjusting for sex, age, calendar time, chronic conditions, and interval between onset and sampling, and applying IPVW, the VE was 18.8% (95% CI: 0.9% to 33.6%) across all age groups. Specifically, the adjusted VE was 45.4% (95% CI: 10.0% to 67.1%) in children aged 0.5–17 years, 43.8% (95% CI: −20.8% to 76.1%) in adults aged 18–59 years, and 8.6% (95% CI: −15.8% to 27.9%) in adults aged 60 years and older.

**FIGURE 4:**
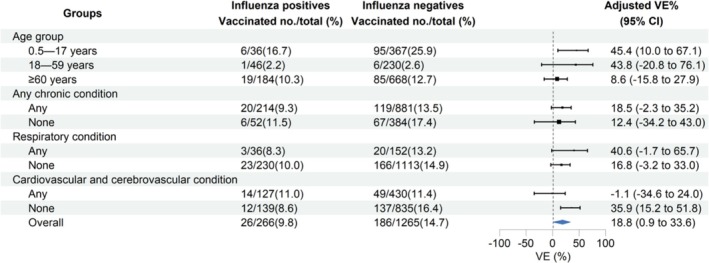
Estimated vaccine effectiveness against influenza A(H1N1)pdm09‐associated hospitalization with severe acute respiratory infection (SARI) in Beijing, China, for the 2024–2025 influenza season. Abbreviations: CI, confidence interval; VE, vaccine effectiveness.

Among patients without chronic conditions, the adjusted VE was 12.4% (95% CI: −34.2% to 43.0%), whereas the adjusted VE among those with chronic conditions was 18.5% (95% CI: −2.3% to 35.2%). The adjusted VE among patients without respiratory conditions was 16.8% (95% CI: −3.2% to 33.0%), while in patients with chronic respiratory conditions, it was 40.6% (95% CI: −1.7% to 65.7%). When comparing patients without and with likely cardiovascular and cerebrovascular conditions, the adjusted VE were 35.9% (95% CI: 15.2% to 51.8%) and −1.1% (95% CI: −34.6% to 24.0%), respectively (Figure 4).

### 3.4 VE Changes by Vaccination Status

**FIGURE 5:**
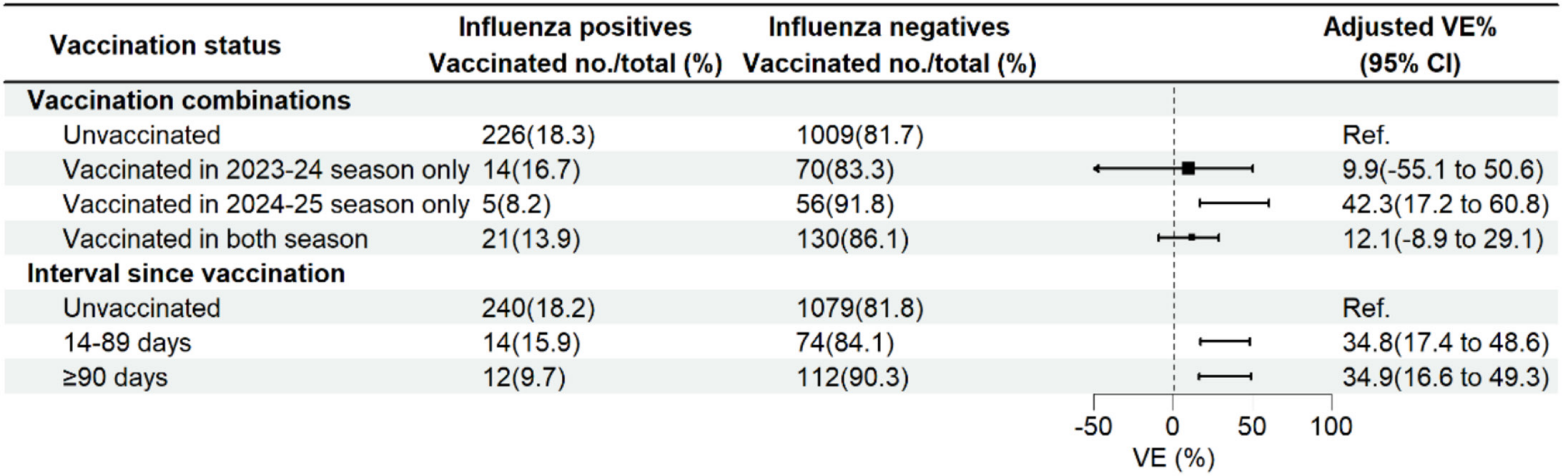
Estimated vaccine effectiveness against influenza A(H1N1)pdm09‐associated hospitalization with severe acute respiratory infection (SARI) among different vaccination statuses in Beijing, China, for the 2024–2025 influenza season. Abbreviations: CI, confidence interval; VE, vaccine effectiveness.

The influenza A(H1N1)pdm09 positive rate was lowest (8.2%) among patients vaccinated only with the 2024–2025 vaccine, followed by 13.9% among those vaccinated in both 2023–2024 and 2024–2025 seasons, 16.7% in those vaccinated only in 2023–2024, and 18.3% in unvaccinated individuals. The adjusted VE against influenza A(H1N1)pdm09 for those who received only the 2024–2025 vaccine, vaccines in both seasons, and only the 2023–2024 vaccine were 42.3% (95% CI: 17.2% to 60.8%), 12.1% (95% CI: −8.9% to 29.1%), and 9.9% (95% CI: −55.1% to 50.6%), respectively. The adjusted VE were 34.8% (95% CI: 17.4% to 48.6%) among patients with a vaccination‐to‐onset interval of 14–89 days and 34.9% (95% CI: 16.6% to 49.3%) among those with an interval of ≥ 90 days (Figure 5).

## 4 Discussion

During the 2024–2025 influenza season, the epidemic primarily occurred from Week 50 of 2024 (December 9th to 15th) to Week 6 of 2025 (February 3rd to 9th), with A(H1N1)pdm09 viruses being the predominant strain in Beijing, China. A suboptimal VE against influenza A(H1N1)pdm09‐associated hospitalization with SARI was observed at 18.8%. The VE point estimate was moderate among children aged 0.5–17 years (45.4%) and younger adults aged 18–59 years (43.8%), whereas it was low among adults ≥ 60 years (8.6%). Furthermore, a low VE was observed among individuals with (18.5%) and without (12.4%) chronic underlying conditions. Specifically, a moderate VE was noted among individuals with respiratory conditions (40.6%).

For the 2024–2025 season, our findings indicate consistent VE estimates for preventing influenza A(H1N1)pdm09‐associated hospitalization in adults aged ≥ 18 years. Our study's estimate of 43.8% (95% CI: −20.8% to 76.1%) closely aligns with the US interim estimate of 39% (95% CI: −14% to 67%) [25]. Conversely, our study identified a comparatively lower VE estimate against A(H1N1)pdm09‐associated hospitalization in individuals under 18 years of age, at 45.4% (95% CI: 10.0% to 67.1%), relative to the US estimate of 63% (95% CI: 30% to 81%) for all children and adolescents aged < 18 years [25]. Interim VE estimates from various European countries ranged from 46% to 53% across all age groups [26], while a German study reported a VE of 57% (95% CI: −10% to 83%) [27], and Denmark documented VE estimates ranging from 36% to 53% for adults aged 65 years and older during the period of October 2024 to March 2025 [28]. These variations across populations and regions may be attributed to differences in vaccination coverage, underlying population immune profiles, or the timing of data collection relative to epidemic peaks.

VE is shaped not only by methodological factors but also, crucially, by the virological context—including the prevalence, timing, and specific strains of the circulating virus. Published studies have revealed both similarities and differences in influenza virus circulation patterns across the aforementioned regions during the study period: peak activity occurred at comparable times (United States: early February 2025 [29]; Europe: Week 6, 2025 [1]; Beijing, China: early January 2025). A(H1N1)pdm09 was the predominant circulating strain, though its proportional distribution varied by region (the United States: approximately 50% [29]; Europe: 66% [30]; Beijing, China: > 99% [4]). For genetic clade distribution, A(H1N1)pdm09 viruses circulating in the United States, Europe, and Beijing, China all belonged to the 6B.1A.5a.2a and/or 6B.1A.5a.2a.1 clades, both of which were well‐matched to the 2024–2025 seasonal vaccine. Specifically, the United States was dominated by clade 5a.2a.1 (67.7%)—the lineage included in the current vaccine—followed by clade 5a.2a (32.3%) [29]. In contrast, clade 5a.2a was predominant in Europe (89%) [31] and the sole circulating clade in Beijing, China [4]. The consistency in viral activity patterns minimizes the confounding effect of transmission intensity on VE comparisons and thereby enhances the overall comparability of the data.

The VE against influenza‐associated hospitalization varies by age group and region. In the United States, interim estimates indicate that the VE against influenza‐associated hospitalization among individuals aged < 18 years (78%) was higher than those aged ≥ 65 years (38% to 57%) and younger adults aged 18–64 years (51%) [25]. Similarly, studies conducted in several European countries during the 2024–2025 season reported VE among children aged 2–17 years (52% to 61%) and younger adults aged 18–64 years (56% to 59%) were higher than adults aged ≥ 65 years and older (38% to 45%) [26]. Interestingly, an interim VE estimates from Germany reported a VE of 69% among individuals aged ≥ 60 years, higher than the overall VE across all age groups (57%) [27]. Similarly, a previous study conducted in Beijing during the 2015–2016 season also reported that VE in adults aged ≥ 60 years (19.5%) was higher than in those aged 5–59 years (−13.9%) and children under 5 years (−63.7%) [32]. Our study found that VE point estimate among adults aged 18–59 years (43.8%) and children aged 0.5–17 years (45.4%) was higher than those among adults aged 60 years and older (8.6%). Variations in VE are influenced by multiple factors, including strain matching, age, preexisting immunity, genetic polymorphisms, and other determinants. Additionally, the presence of chronic underlying conditions may compromise influenza vaccine responsiveness [33]. Generally, influenza vaccines tend to be less effective in the elderly compared to young adults, as the aging immune system typically results in a diminished vaccine response in this demographic [34]. To overcome this limitation, high‐dose trivalent inactivated influenza vaccines and adjuvanted vaccines have been developed and used in older adults in many countries [28, 35]. Studies have shown that these vaccines are more immunogenic and effectiveness than standard‐dose vaccines in preventing influenza virus infections and their complications in older adults [36, 37, 38]. To improve the VE in the elderly population, China may consider promoting the use of the above‐mentioned vaccines among the elderly.

We also evaluated VE analyses on individuals with underlying chronic diseases. The results showed that individuals with chronic underlying conditions had a low VE (point estimate) of 18.5%; however, those with respiratory conditions had a moderate VE (40.6%). These findings are consistent with previous study. For instance, a cohort study using the Taiwan Longitudinal Health Insurance Database from 1996 to 2008 found that elderly patients with COPD who received influenza vaccinations had a significantly lower risk of hospitalization for acute coronary syndrome (HR = 0.46, 95% CI: 0.39% to 0.55%) [39]. Although the study did not directly test for influenza infection, its central hypothesis was that vaccination reduces the risk of influenza infection, thereby indirectly lowering the risk of influenza‐associated ACS.

Immunity acquired through influenza virus infection or vaccination diminishes over time, with the extent of this decline influenced by factors such as age, physical condition, and the specific vaccine antigen. Typically, antibody levels peak within 2–3 weeks postvaccination and decrease gradually thereafter [40]. Multiple studies have provided evidence supporting this phenomenon. For instance, research conducted among hospitalized patients with acute respiratory illness indicated that the highest VE was observed shortly after vaccination, followed by an absolute decline in VE of approximately 8%–9% per month postvaccination [31]. However, our study revealed that VE against influenza A(H1N1)pdm09‐associated hospitalization was similar within the first 3 months postvaccination compared to the period of 3 months or more after vaccination. This finding contrasts with previous studies that reported a waning VE over time and may be explained by the high proportion of elderly participants in our study. Given their typically lower immune response, these individuals may not have generated sufficient antibody levels following vaccination. Consequently, the decline in vaccine efficacy over time was not significantly observed in this cohort. Additionally, the limited sample size could have contributed to the lack of significant findings.

This study was subject to several limitations. First, although this study represents one of the most extensive investigations in Beijing assessing VE against A(H1N1)pdm09 in hospitalized patients, the suboptimal vaccination coverage during the 2024–2025 season—particularly among adults aged 18–59 years—and the limited incidence of breakthrough infections resulted in data sparsity within subgroup analyses. In several subgroups, the exceedingly low number of vaccinated cases, coupled with multiple adjustment variables, may have introduced sparse data bias and compromised model stability, thereby diminishing the reliability of subgroup‐specific VE estimates. Consequently, these findings should be regarded as exploratory. Nonetheless, the primary analysis of the overall population was conducted with an adequate sample size, yielding more robust VE estimates. Secondly, discrepancies were noted between the VE point estimates stratified by underlying chronic conditions and both the overall and age‐stratified VE estimates. This is likely due to the overrepresentation of adults aged ≥ 60 years within the chronic condition subgroup, which impedes a comprehensive characterization of the independent effect of chronic conditions. Third, unmeasured or residual confounding may persist despite the application of inverse probability weighting (IPW) and adjustment for measured confounders. Fourth, while the utilization of vaccination data obtained from an authoritative database enhanced the precision of exposure assessment and reduced reporting bias, potential discrepancies due to errors in the collection of demographic information could result in the misclassification of vaccination status. Specifically, when participant information was used to query the vaccination database to verify vaccination status, errors in the collected information made it significantly more likely for vaccinated individuals to be misclassified as unvaccinated than the reverse. In the context of nondifferential misclassification, this systematic bias would ultimately lead to an underestimation of VE. Additionally, the use of questionnaires to collect information on chronic conditions and onset dates may introduce reporting bias.

In conclusion, during the 2024–2025 influenza season, the VE against influenza A(H1N1)pdm09‐associated hospitalizations with SARI was suboptimal. However, a moderate VE was observed among individuals aged 0.5–17 years and younger adults aged 18–59 years, whereas VE remained low in those aged 60 years and older. Additionally, a moderate VE was observed among individuals with preexisting respiratory conditions. To alleviate the impact of influenza‐associated hospitalizations, enhanced vaccination strategies are warranted, including the potential introduction of high‐dose or adjuvanted influenza vaccines for the elderly population in China.

## Author Contributions

**Chunna Ma:** conceptualization, software, data curation, writing – original draft. **Ying Sun:** conceptualization, data curation, funding acquisition. **Jiaxin Ma:** data curation. **Yingying Wang:** data curation. **Xiaodi Hu:** data curation. **Ying Shen:** validation, writing – review and editing, supervision. **Li Zhang:** software, data curation. **Jiaojiao Zhang:** data curation. **Lu Zhang:** data curation. **Wei Duan:** validation, data curation. **Jia Li:** data curation. **Quanyi Wang:** formal analysis, project administration. **Daitao Zhang:** formal analysis, supervision, project administration, funding acquisition. **Peng Yang:** writing – review and editing, formal analysis, supervision, project administration, funding acquisition.

## Funding

This work was supported by Beijing Natural Science Foundation grant L232014 (Y.S.), Capital's Funds for Health Improvement and Research grant 2022‐1G‐3014 (P.Y.), High Level Public Health Technical Talent Training Plan grant xuekedaitouren‐01‐03 (P.Y.), and Beijing Research Center for Respiratory Infectious Diseases Project grant BJRID2025‐005 (D.T.Z.).

## Ethics Statement

The study was approved by the Institutional Review Board and Human Research Ethics Committee of Beijing Center for Disease Prevention and Control (No. 2017 (2)).

## Consent

Informed consent was obtained from all subjects involved in the study.

## Conflicts of Interest

The authors declare no conflicts of interest.

## Data Availability

The original database, which contains confidential patient information, cannot be made publicly accessible. The anonymized data used in this study are available based on reasonable request to the corresponding author.
